# Development and Evaluation of NanoPCR for the Detection of Goose Parvovirus

**DOI:** 10.3390/vetsci9090460

**Published:** 2022-08-27

**Authors:** Haoyuan Ma, Xu Gao, Jingfeng Fu, Haowen Xue, Yanhao Song, Kunru Zhu

**Affiliations:** Laboratory for Animal Molecular Virology, Department of Veterinary Medicine, College of Agricultural, Yanbian University, Yanji 133002, China

**Keywords:** goose parvovirus, nanoparticles, nanoPCR, VP3 gene, epidemiology

## Abstract

**Simple Summary:**

Gosling plague (GP), an acute, virulent infectious disease caused by goose parvovirus (GPV), is a serious problem for livestock and poultry breeding. At present, there is no effective treatment available. The disease is vertically transmitted in geese, and some infected pregnant females are often recessive carriers of the virus, making it very difficult for farmers to detect GPV in the clinical setting. Although there are many clinical testing methods for GPV, some of them still suffered from shortcomings such as being time-consuming and labor-intensive. In this study, gold nanoparticles were put into a conventional PCR reaction system, and the first nanoPCR reaction was successfully established to detect infected GPV in the clinic, thus providing a practical method for the detection of GPV clinical infection.

**Abstract:**

Gosling plague (GP) is an acute and hemorrhagic infectious disease caused by goose parvovirus (GPV). The goose industry suffers significant economic losses as a result of GP, which is found to be widespread worldwide, with high rates of morbidity and mortality. Our group developed a novel technique for detecting GPV nanoparticle-assisted polymerase chain reaction (nanoPCR) and the characterization of its specificity and sensitivity. It was developed by using the traditional polymerase chain reaction (PCR) and nanoparticles. The findings of this study revealed that GPV nanoPCR products were 389 bp in length, and the lower limit of the nanoPCR assay was 4.68 × 10^2^ copies/μL, whereas that of the conventional PCR assay was 4.68 × 10^4^ copies/μL. A total of 230 geese suspected of GPV were detected using nanoPCR, with a positive rate of 83.0% and a specificity of 73%, respectively. Overall, we present a hitherto undocumented method for identifying GPV by using nanoPCR to aid in the evaluation of subclinical illness.

## 1. Introduction

Gosling plague (GP) is an infectious disease caused by the goose parvovirus (GPV), which was first discovered in 1956 by a Chinese scientist, Dingyi Fang, who isolated the virus in 1962 [1]. In the aftermath, the disease was named Derzsy’s disease and has been identified worldwide. GPV epidemics were documented around the world between the 1950s and the 1980s. The clinical signs of brain atrophy, weight loss, weakness, and severe diarrhea occur in geese under ten days of age and muscovy ducks older than two weeks [2]. The infected geese exhibit fibrinous and necrotizing enteritis and small intestinal embolism, which are formed when the surface of the intestinal mucosa dies and falls off [3].

GPV is mainly transmitted through the secretions and excreta of sick geese [4]. The pathological features of GPV are characterized by surface layer necrosis and the peeling of the small intestinal mucosa, and fibrous exudates coagulating into emboli or a pseudomembrane wrapping around the intestinal contents [5,6]. It is important to note that GPV can be transmitted vertically, which makes it possible for recessive adult geese with GPV to transmit the infection to goslings through the cloaca, complicating disease control [7].

GPV is a member of the *Parvovirus* genus within the *Parvoviridae* family, with a genome consisting of 5106 nucleotide-long, single-stranded DNA. The coding region is split into non-structural proteins NS1 and NS2 [8,9], and structural proteins VP1, VP2, and VP3 [10,11,12]. NS1 and NS2 are important for early viral replication and gene expression regulation in GPV. However, GPV’s capsid can adsorb cells, which significantly impacts the virus’s pathogenicity and virulence. VP3 encodes the primary structural protein and is the most conservative and stable of the viral capsid proteins [13,14]. Therefore, it is often used as a target gene in genetic engineering.

Nanoparticle-assisted polymerase chain reaction (nanoPCR) incorporates gold nanoparticles with diameters of less than 100 nm into a standard PCR solution to form a “nanoPCR” reaction solution [15,16,17]. This technique requires no specialized or sophisticated instruments. In biology, gold nanoparticles are referred to as “colloid gold” [18]. Research has shown that gold nanoparticles’ high thermal conductivity allows PCR procedures to reach the necessary reaction temperature more quickly. NanoPCR performed well in distinguishing mismatch primers, and only 1 nm gold nanoparticles were revealed to be effective in preventing mismatching, facilitating the development of nanoPCR in genetics [19]. Additionally, the sensitivity and specificity were 100–1000 times greater than conventional PCR [20]. Thus far, multiple studies have demonstrated the efficacy of nanoPCR. NanoPCR has been used to identify preclinical infections such as porcine parvovirus (PPV) [21], bovine respiratory syncytial virus (BRSV) [22], and human papillomavirus (HPV) [23]. However, to the best of our knowledge, the application of nanoPCR detection in GPV has not been used.

Laboratory diagnostic approaches, such as enzyme-linked immune sorbent assay (ELISA) [24], PCR [25], quantitative PCR (qPCR) [26], etc., have been increasingly common in the clinical identification of GP since its occurrence. In terms of detection, some experimental approaches still had drawbacks, such as being time-consuming, labor-intensive, and having poor specificity, which made them difficult to use in clinical practice. Therefore, it is necessary to develop a nanoPCR for the detection of GPV in goslings. This study intends to establish GPV nanoPCR and determine its clinical detection effect by comparing it with conventional PCR. This will have significant repercussions for the clinical prevention and treatment of GP infectious diseases.

## 2. Materials and Methods

### 2.1. Viruses and Clinical Samples

The GPV YBYJ strain, goose paramyxovirus (GPMV), duck plague virus (DPV), and muscovy duck parvovirus (MDPV) were all preserved by our laboratory. In total, 230 clinical samples were collected from non-immune geese suspected of GPV infection in some goose farms in Jilin Province.

### 2.2. The Extraction of DNA/RNA from the Samples

Viral genomic DNA/RNA extraction kits (CWBIO, Taizhou, China) were used to extract GPV, DPV, MDPV, and GPMV, and they were kept at −20 °C. A reverse transcription kit (TaKaRa, Dalian, China) was used to synthesized cDNA (GPMV), and it was kept at −80 °C.

### 2.3. Primer Design and Plasmid Construction

The whole-genome sequences of GPV, nGPV, and MDPV were downloaded from GenBank, and the VP3 gene was found and compared in DNASTAR (LaserGene, USA). The highly conserved region of the GPV-VP3 genome was determined via alignment and by using the Oligo 6 software (Molecular Biology Insights, USA) to design primer pairs GPV-VP3-F1/R1, TaqMan real-time primer pairs GPV-VP3-F3/R3, and TaqMan probe GPV-P (Table 1).

The 1603 bp GPV-VP3 gene was derived from the GPV-YBLJ strain (GenBank accession no. JN836326.1) and amplified by the GPV-VP3-F2 and GPV-VP3-R2 primers in 20 μL (TaKaRa, Dalian, China) at 94 °C for 5 min using pre-denaturation, followed by 30 cycles at 94 °C for 30 s, 53 °C for 1 min, 72 °C for 2 min, and finally 72 °C for 10 min. The reaction was performed on a PCR machine (Bio-Rad, Hercules, CA, USA). The entire sequence of the GPV-VP3 gene was cloned into the plasmid vector pMD19-T (TaKaRa, Dalian, China) and multiplied in DH5α (TransGen, Beijing, China). After purification, a plasmid mini kit (Omega, USA) was used to extract the recombinant pMD19-T-GPV-VP3 (copy quantity: 4.68 × 10^10^). PCR products were confirmed by sequencing.

### 2.4. Optimization of the Reaction Conditions of nanoPCR

pMD19-T-GPV-VP3 was used as a standard for the nanoPCR assay for the detection of GPV. The GPV-VP3-F1 and GPV-VP3-R1 primers were used for amplification. In order to optimize the nanoPCR reaction system, the nanoparticle diameter, nanoparticle concentration, annealing temperature, template concentration, and primer concentration were optimized. NanoPCR was carried out in a 25 μL reaction volume containing nanoparticles (Jieyi, Shanghai, China) of varying diameters (10, 15, 20, 30, and 40 nm). We progressed from nanoparticles with a volume of 0.6 μL to nanoparticles with a volume of 3.0 μL after determining the nanoparticle diameter range. The annealing temperature in the PCR machine was 50 to 60 °C, the plasmid template volume was 0.1 to 1.4 μL, and the primer volume (10 μM) was 0.1 to 1.0 μL. The PCR amplification scheme was pre-denatured at 94 °C for 5 min, followed by 30 cycles of 94 °C for 30 s, 50 to 60 °C for 30 s, 72 °C for 30 s, and a final extension at 72 °C for 5 min. A healthy (non-GPV-infected) gosling was used as a negative control. The final product was electrophoresed on 1% agarose gels.

### 2.5. Sensitivity, Specificity, and Reproducibility Tests of nanoPCR

The pMD19-T-GPV-VP3 plasmid was used as a reference, and PCR amplifications were performed after serial dilutions of 10 times, according to the optimal size, concentration, and reaction conditions of the nanoPCR assay. Amplification was performed using the GPV-VP3-F1 and GPV-VP3-R1 primers. We then compared the detection limits of nanoPCR and conventional PCR. ddH_2_O was used as a negative control. Three separate experiments were conducted at different times by different people to allow the experiments to be replicated.

Meanwhile, the specificity of the GPV nanoPCR was tested using genomic DNA from the GPMV, DPV, and MDPV viruses, as well as ddH_2_O as a negative control. The product was analyzed via 1% agarose gel electrophoresis.

### 2.6. Detection of GPV in Clinical Samples by nanoPCR

NanoPCR, TaqMan real-time PCR, and conventional PCR were used to screen stool swabs or nasal swabs collected from 230 samples suspected of GPV infection in Yanbian Korean Autonomous Prefecture from 2019 to 2022. Genomic template extraction was performed on all samples. The swabs were stored in PBS (1:5) and evenly squeezed on the wall of the centrifuge tube, freeze–thawed 3 times, centrifuged at 4000 r/min for 30 min, and stored at −20 °C for later use.

The TaqMan real-time PCR detection was carried out according to the reaction system optimized in the previous stage of our research group [27]. The positive samples were subjected to sequencing. A phylogenetic tree was constructed to compare and assess the published sequences, including nGPV and MDPV, to determine the specificity of nanoPCR and the current prevalence of GPV in China. Summary statistics for these sequences are shown in Table S1. The MEGA 6.0 software (Mega Limited, Auckland, New Zealand) utilized the maximum likelihood method (Poisson model) and 1000 bootstrap repetitions to perform a phylogenetic analysis.

## 3. Results

### 3.1. Optimization of nanoPCR

**Figure 1:**
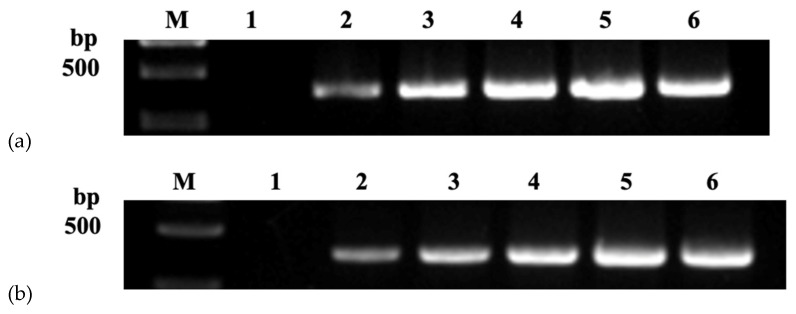
Optimization of diameter (**a**) and concentration (**b**) of nanoparticles: (**a**) Lane M, DL 2000 Marker; Lane 1, negative control; Lanes 2–6, 10, 15, 20, 30, and 40 nm; (**b**) Lane M, DL 2000 Marker; Lane 1, negative control; Lanes 2–6, 0.1, 0.2, 0.3, 0.4, and 0.5 mM.

For nanoPCR, the diameter and concentration of nanoparticles were optimized. When the concentration was 0.4 mM, and the diameter was 30 nm, the optimal effect and bands were obtained (Figure 1).

**Figure 2:**
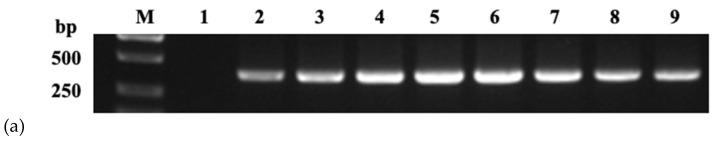

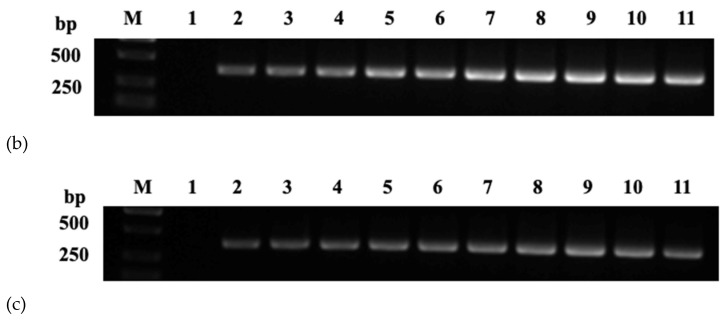
Optimization of annealing temperature (**a**), primer concentration (**b**), and template concentration (**c**): (**a**) Lane M, DL 2000 Marker; Lane 1, negative control; Lanes 2–9,50.0, 50.7, 51.9, 53.7, 56.1, 58.0, 59.2, and 60.0 °C; (**b**) Lane M, DL 2000 Marker; Lane 1, negative control; Lanes 2–11, 0.05, 0.10, 0.15, 0.20, 0.25, 0.30, 0.35, 0.40, 0.45, and 0.50 μM; (**c**) Lane M, DL 2000 Marker; Lane 1, negative control; Lanes 2–11, 2, 4, 8, 12, 14, 16, 18, 20, 24, and 28 ng.

The F1 and R1 primers were used to establish the reaction between nanoPCR and conventional PCR. For the best results, all conditions were standardized. The final product was 389 bp, the ideal primer concentration was 0.40 μM (0.8 μL), the plasmid was 20 ng (1.0 μL), and the annealing temperature was 53.7 °C, according to the data (Figure 2).

### 3.2. Sensitivity of nanoPCR

**Figure 3:**
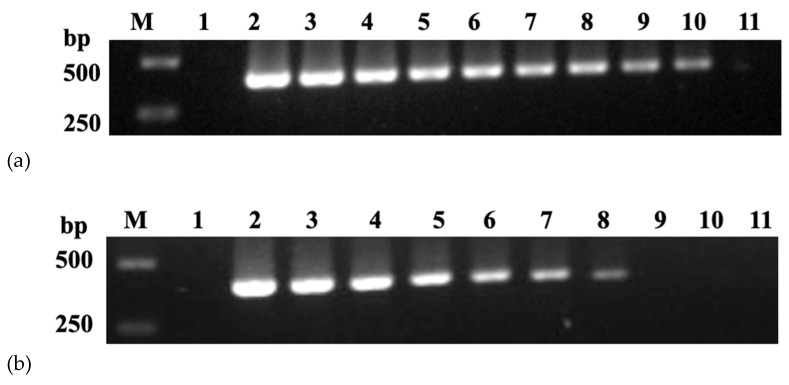
The sensitivity of nanoPCR (**a**) and conventional PCR (**b**): (**a**) Lane M, DL 2000 Marker; Lane 1, negative control; Lanes 2–11, 4.68 × 10^10^, 4.68 × 10^9^, 4.68 × 10^8^, 4.68 × 10^7^, 4.68 × 10^6^, 4.68 × 10^5^, 4.68 × 10^4^, 4.68 × 10^3^, 4.68 × 10^2^, and 4.68 × 10^1^ copies/μL; (**b**) Lane M, DL 2000 Marker; Lane 1, negative control; Lanes 2–11, 4.68 × 10^10^, 4.68 × 10^9^, 4.68 × 10^8^, 4.68 × 10^7^, 4.68 × 10^6^, 4.68 × 10^5^, 4.68 × 10^4^, 4.68 × 10^3^, 4.68 × 10^2^, and 4.68 × 10^1^ copies/μL.

In order to test the sensitivity of nanoPCR, different quantities of plasmids were used as positive templates for conventional PCR and nanoPCR reactions. The standard concentration of the pMD19-T-GPV-VP3 plasmid was 4.68 × 10^10^ copies/μL, and the concentration was serially diluted to 4.68 copies/μL. The results showed that the detection limit of nanoPCR was 1/100 that of the conventional PCR assay (Figure 3). The same results were obtained by different operators.

### 3.3. Specificity of nanoPCR

**Figure 4:**
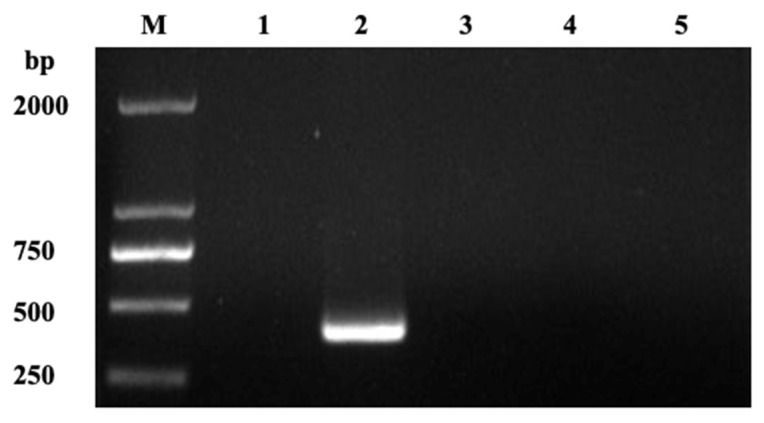
The specificity of nanoPCR. Lane M, DL 2000 Marker; Lane 1, negative control; Lanes 2–5, GPV, MDPV, DPV, and GPMV.

According to the gel electrophoresis results, nanoPCR could specifically amplify GPV but not GMPV, DPV, or MDPV (the concentration was 20, 18, 22, and 25 ng/μL), suggesting that nanoPCR has excellent specificity for the detection of GPV (Figure 4).

### 3.4. GPV Detection in Clinical Samples and Phylogenetic Analysis

**Figure 5:**
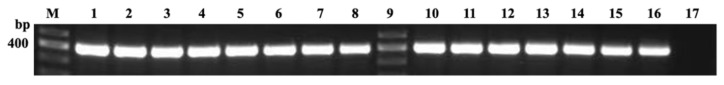
Detection results of GPV nanoPCR in clinical samples. Lane M and 9, DL 1000 Marker; Lane 1–8 and 10–16, GPV clinical samples; Lane 17, healthy gosling.

The swabs from 230 samples suspected of GPV infection were tested using nanoPCR, TaqMan real-time PCR, and conventional PCR (Figure 5). Among the 230 samples analyzed, 191 samples tested positive using both nanoPCR and TaqMan real-time PCR (83.0%), and 174 samples were positive using conventional PCR (75.6%). Approximately 14 samples were negative using the conventional PCR assay but positive using nanoPCR, with a relative specificity of 73% (Table S2). However, no nanoPCR-negative samples were found to be positive using conventional PCR. For clinical samples, both nanoPCR and TaqMan real-time PCR had superior specificity and sensitivity to conventional PCR. Based on the sequence analysis, it was found that the nanoPCR amplification products and the reference GPV sequences were identical.

**Figure 6:**
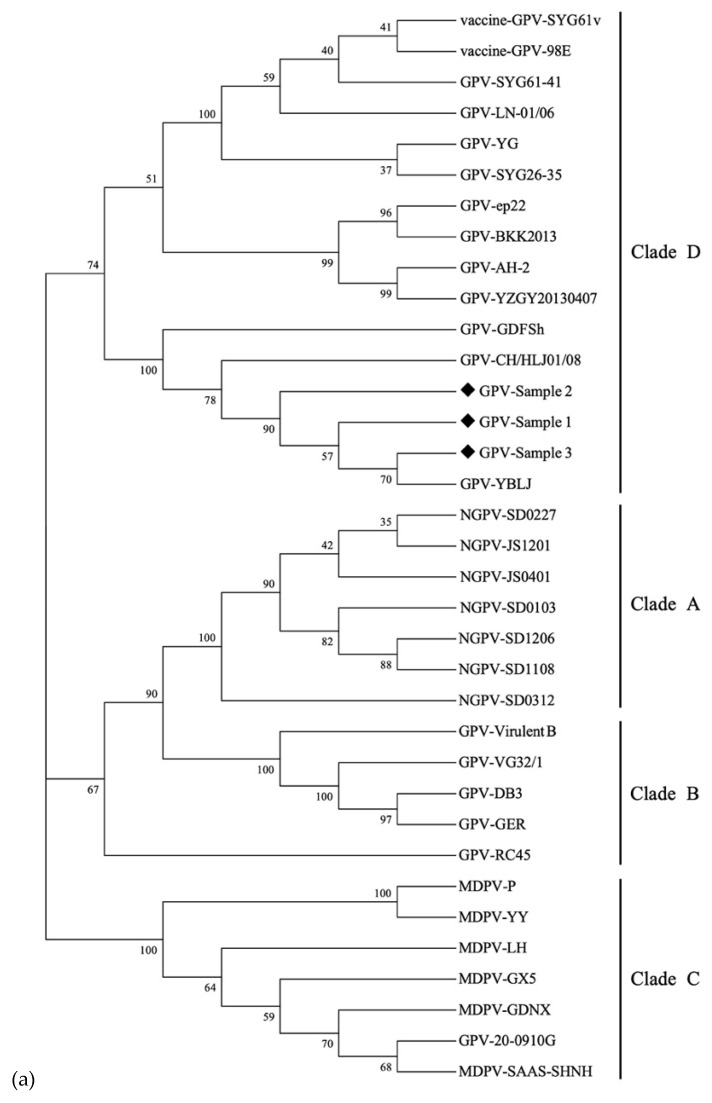

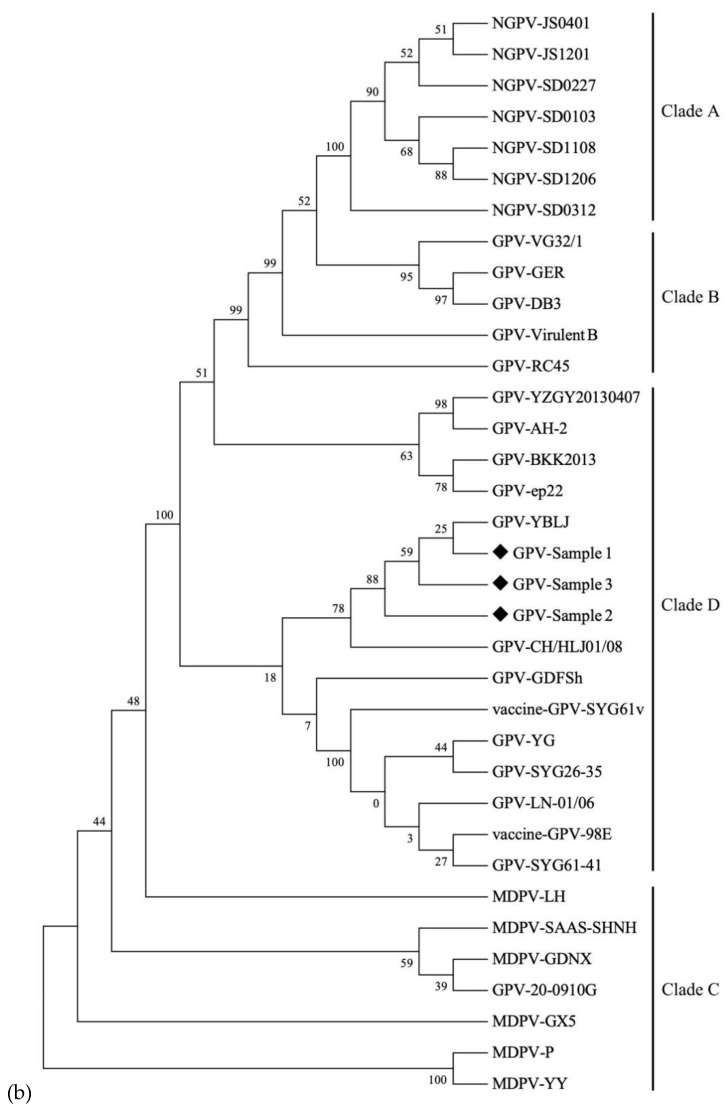
Phylogenetic analysis of GPV with other viruses based on the VP3 nucleotide sequences: (**a**) neighbor-Joining (N–J) method; (**b**) maximum likelihood (M–L) method. The phylogenetic tree was based on the complete VP3 sequences of GPV, together with GPV, nGPV, and MDPV. Nucleotide sequences were analyzed using the MEGA 6.0 software with a bootstrap test of 1000 replicates. The clades were used to differentiate between the different strains. The substitution model was HKY + G. The diamond-shaped icons (◆) were the clinical samples in this study.

Phylogenetic trees were constructed using 32 complete VP3 gene sequences of GPV, MDPV, and nGPV from GenBank (Figure 6). The results showed that the cases collected from goose farms in Jilin Province were closely related to GPV-YBLJ, GPV-CH/HLJ01/08, and GPV-GDFsh, suggesting they were from the same cluster. However, there are still GPV strains isolated in China that do not belong to the same evolutionary branch as the clinical sample, implying that the GPV strains in China have genetic variations. Some of the sequences in the clinical trials have been aligned with the sequences of nGPV and MDPV obtained from GenBank (Figure S1). Nonetheless, there is currently no epidemic nGPV variant in this region.

## 4. Discussion

The frequency of GPV reports from mainland China, Chinese Taiwan [28], Turkey [29], and other Asian countries [30,31] has increased since GP was identified in China four decades ago. GPV poses a threat to the gosling and geese industries [32]. Despite the availability of commercial vaccines and high-immune serum [33], there are still fatalities associated with GPV-related deaths. The difficulty of early clinical diagnosis in the breeding sector is further compounded by the fact that the GPV can disguise itself as a concealed virus carried by geese after an outbreak [34,35]. GPV must be detected quickly and accurately so that it can be diagnosed and prevented.

The traditional laboratory diagnosis of GPV infection uses serological, pathological, and molecular methods. In previous studies, Kisary employed goose and muscovy embryos to isolate and identify GPV [36]. Despite this, early clinical diagnosis is impossible due to the inconvenience of the experimental setting and the complexity of the procedure. To detect GPV, She et al. developed an agar diffusion method [37]. Due to its low sensitivity and specificity, it was not suitable for mixed infection samples. Gene amplification technology is a popular method for the early detection of GP. To detect GPV, LAMP, SYBR Green I, LC Green, and TaqMan probes are commonly employed [38,39,40]. Due to these limitations, it is difficult to promote the aquaculture clinical diagnosis with these approaches, which often involve complicated primer designs and expensive equipment and are susceptible to template contamination. For the early detection of the disease in animals, conventional PCR can be employed [41,42,43], although its sensitivity and specificity are significantly lower than real-time PCR. Traditional PCR has not been widely used due to poor specificity and sensitivity. In this study, gold nanoparticles were added to the PCR system as a means of developing a very sensitive and specific nanoPCR method for the detection of GPV.

The nanoPCR method has been used to detect parvoviruses such as PPV [21], mink enteritis virus (MEV) [44], and canine parvovirus (CPV) [45]. NanoPCR has been widely used and supported by farmers. However, a GPV nanoPCR assay has yet to be developed. Using a conventional plasmid and 10-fold dilution, nanoPCR amplification was used to amplify the highly conserved portion of the GPV-VP3 gene. When compared with immunochromatography combined with colloidal gold particles, the detection performance of nanoPCR was greater because it could distinguish between viral infections of various types [46]. NanoPCR, for example, is 100 times more sensitive than conventional PCR, so it might be useful for detecting early infections. It was shown in this experiment that the detection limits of nanoPCR were 100 times more sensitive than those of conventional PCR in terms of detection. However, it was discovered throughout the clinical experiment that the TaqMan real-time PCR technology had high sample requirements and that even a small amount of pollution might cause errors in the detection of the samples [47]. Therefore, this approach is not suitable for clinical testing.

In Jilin Province, an agricultural region in northeast China, chicken products are in high demand. Poultry farming is also a significant source of income in this region. The adoption of nanoPCR is critical for preventing GPV prevalence and infection. This experiment was carried out on 230 samples from different goose farms in Yanbian Korean Autonomous Prefecture, Jilin Province, suspected of being infected with GPV. A total of 191 positive samples were found, with an 83.0% GPV detection rate. The conventional PCR assay yielded a 75.6% positive result rate for GPV. Based on gene sequencing, sequence alignments, and phylogenetic trees, we found that the detection success rate of nanoPCR was quite high. As evidenced by these findings, nanoPCR can be used in clinical settings.

In the current study, we developed GPV-VP3 specific primers for nanoPCR detection by comparing the GPV genomic sequences in GenBank and screening its conservative sequence region, which is 100 times more sensitive than conventional PCR. The ideal nanoparticle volume was 2.4 μL, with a diameter of 30 nm. Most importantly, this is the first report on nanoPCR technology for GPV detection. In this study, we explored how nanoPCR was introduced as a technique that can be used in virology.

## Figures and Tables

**Table 1:** The primers used in this study.

Primers	Primer Sequences (5′–3′)	Product Size (bp)
GPV-VP3-F1	CCTGGACCAGAGAGTTAGGGCCTAT	386
GPV-VP3-R1	TCTGCCAAACCATTCCTGGTAAAGC	
GPV-VP3-F2	CTCGAGATGGCAGAGGGAGGAG	1605
GPV-VP3-R2	CGGTCGACTTACAGATTTTGAGTTAG	
GPV-VP3-F3	CAACCATTGGGGAATCAGAC	121
GPV-VP3-R3	TTGAATTGTTGACGTGAGATTGT	
GPV-P	FAM-TCTGATCCTGCGTTGTGACTTCTTTG-BHQ1	

## Data Availability

The data that supports the findings of this study are available in the supplementary material of this article.

## Supplementary Materials

The following supporting information can be downloaded at: https://www.mdpi.com/article/10.3390/vetsci9090460/s1, Figure S1: Nucleotide sequence alignment of clinical samples, nGPV, and MDPV sequences; Table S1: The GPV-VP3 sequences and the GenBank accession numbers were used in this investigation; Table S2: Statistics on GPV infection in Yanbian Korean Autonomous Prefecture from 2019 to 2022.
