# Supplementary material for: Development and Evaluation of NanoPCR for the Detection of Goose Parvovirus
Source: Vet Sci. 2022 Aug 27;9(9):460. doi: 10.3390/vetsci9090460 (PMC9506417; doi:10.3390/vetsci9090460)
Supplement: Supplementary file 1 [file vetsci-09-00460-s001.zip › vetsci-1844459-supplementary.pdf]

Supplemental material

Development and Evaluation of NanoPCR for the Detection of Goose Parvovirus

Haoyuan Ma, Xu Gao, Jingfeng Fu, Haowen Xue, Yanhao Song and Kunru Zhu

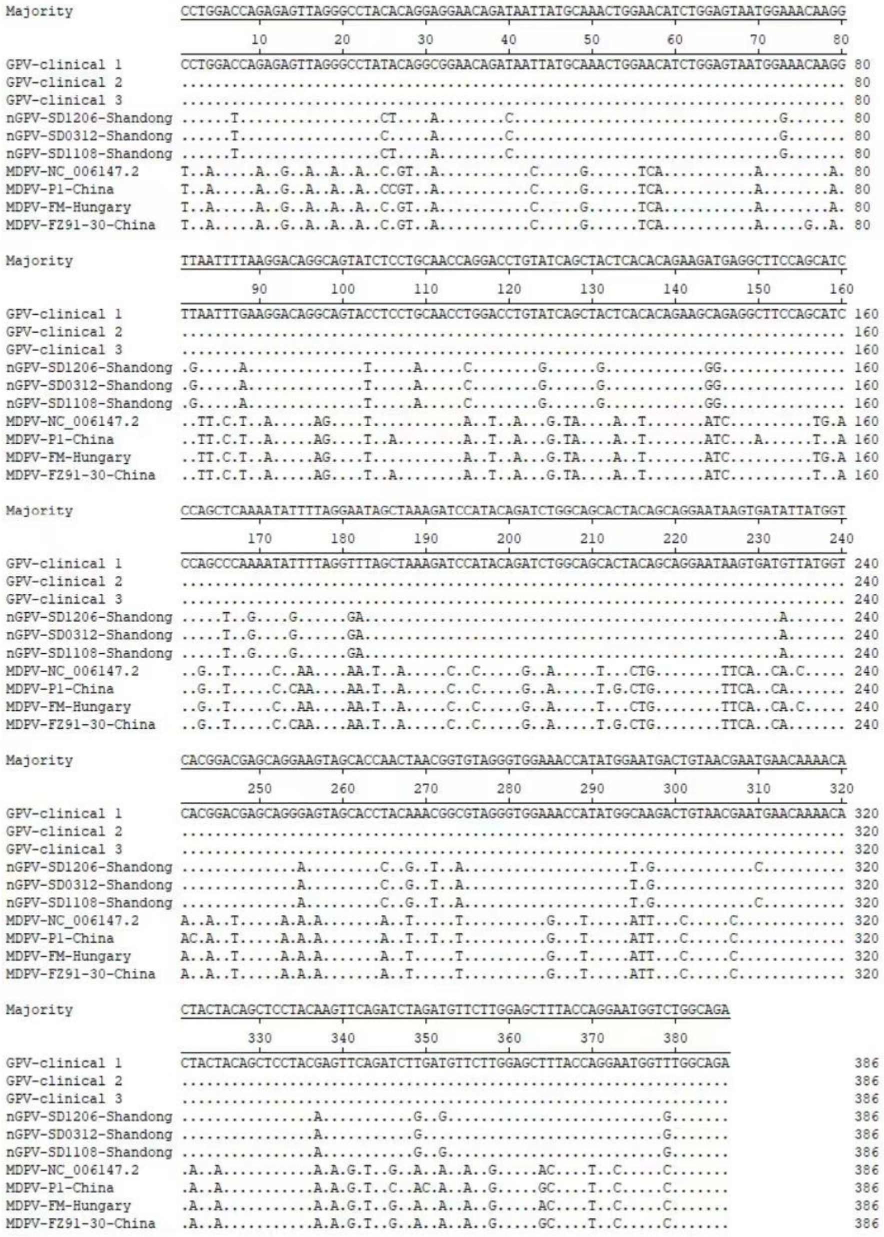

Figure S1. Nucleotide sequence alignment of clinical samples, nGPV, and MDPV sequences.

Table S1. The sequences used in this study and the GenBank accession numbers.

Strains	Accession No.	Genetic type	Host	Submitted year	Origin
YBLJ	JN836326	GPV	Goose	2012	China
SYG61v	KC996729	GPV	Goose	2015	China
GPV-98E	KT598506	GPV	Goose	2016	China
SYG61-41	DQ299421	GPV	Goose	2005	China
GDFsh	EU088103	GPV	Goose	2007	China
GPV/CHHLJ01/08	FJ240170	GPV	Goose	2008	China
YGZY20130407	KP053644	GPV	Goose	2015	China
AH-2	KU168321	GPV	Goose	2016	China
BKK2013	LC651623	GPV	Goose	2021	Thailand
Virulent B	U25749	GPV	Goose	1995	Hungary
GPV GER	KU684472	GPV	Ornamental duck	2016	Poland
GPV-YG	AF416726	GPV	Goose	2001	China
VG32/1	EU583392	GPV	Goose	2008	China
LN-01/06	EU218524	GPV	Goose	2007	China
DB3	EU088102	GPV	Goose	2007	China
20-0910G	OK392126	GPV	Goose	2022	China
RC54	MH717784	GPV	Goose	2019	China
SYG26-35	KR265067	GPV	Goose	2015	China
ep22	GQ392034	GPV	Goose	2010	China
MDPV-GX5	KM093740	MDPV	Muscovy duck	2015	China
LH	KY069274	MDPV	Mule duck	2017	China
GDNX	MH204100	MDPV	Muscovy duck	2018	China
SAAS-SHNH	KC171936	MDPV	Muscovy duck	2013	China
P	KU844281	MDPV	Muscovy duck	2016	China
YY	KX000918	MDPV	Muscovy duck	2016	China
JS0401	MN172360	nGPV	Cherry Valley duck	2020	China
JS1201	MN172361	nGPV	Cherry Valley duck	2020	China
SD0103	MN172362	nGPV	Cherry Valley duck	2020	China
SD0227	MN172363	nGPV	Cherry Valley duck	2020	China
SD0312	MN172364	nGPV	Cherry Valley duck	2020	China
SD1108	MN172365	nGPV	Cherry Valley duck	2020	China
SD1206	MN172366	nGPV	Cherry Valley duck	2020	China

Table S2. Statistics on GPV infection in Yanbian Korean Autonomous Prefecture from 2019 to 2022.

Year	Age	Number	Sample	Place	GPV rate
2019	5-d	20	Fecal	Longjing	(20/20)100%
	5-d	10	Fecal	Wangqing	(10/10)100%
	9-d	10	Fecal	Yanji	(7/10)100%
	16-d	14	Fecal	Antu	(8/14)57.1%
	18-d	16	Fecal	Longjing	(12/16)75%
2020	11-d	10	Fecal	Yanji	(10/10)100%
	8-d	13	Fecal	Longjing	(8/13)61.5%
	20-d	15	Fecal	Hunchun	(4/15)26.7%
	6-d	12	Nasal	Dunhua	(12/12)100%
2021	3-d	10	Nasal	Longjing	(10/10)100%
	5-d	20	Fecal	Hunchun	(18/20)90%
	7-d	20	Fecal	Yanji	(20/20)100%
	11-d	30	Nasal	Wangqing	(25/30)83.3%
	16-d	10	Fecal	Yanji	(10/10)100%
2022	5-d	5	Fecal	Longjing	(5/5)100%
	7-d	10	Nasal	Longjing	(9/10)90%
	14-d	5	Fecal	Longjing	(3/5)60%
